# Extent of Resection in Meningioma: Predictive Factors and Clinical Implications

**DOI:** 10.1038/s41598-019-42451-z

**Published:** 2019-04-11

**Authors:** Jean-Michel Lemée, Marco V. Corniola, Michele Da Broi, Holger Joswig, David Scheie, Karl Schaller, Eirik Helseth, Torstein R. Meling

**Affiliations:** 1 Department of Clinical Neurosciences, Division of Neurosurgery, Geneva University Hospitals, Genève, Switzerland; 2 Faculty of Medicine, University of Oslo, Oslo, Norway; 3 Section of Neuropathology, Rigshospitalet, Copenhagen, Denmark; 4 Faculty of Medicine, University of Geneva, Genève, Switzerland; 5 Department of Neurosurgery, Oslo University Hospital, Oslo, Norway

## Abstract

Meningiomas present as intracranial extra-axial lesions with dural attachment, which are primarily managed surgically. The extent of resection (EOR) may vary depending on patient- and tumor-related factors. The aim of this study is to identify preoperative predictive factors of EOR and to propose an estimation of the predicted gross total resection (GTR) based of patient- and tumor-characteristics. 1469 patients from a retrospectively (1990 to 2002) and prospectively managed (2003 to 2010) databank of Oslo University Hospital, Norway, totalling 11,414 patient-years of follow-up were included. Patients had a mean age at surgery of 64 ± 20.1 years with a female-to-male ratio was 2.4:1 and a mean KPS of 81.2 ± 12.1. Skull-base meningiomas represented 47% of all cases. WHO grades were I in 92.3%, II in 5.2%, and III in 2.2%. Bone infiltration was described in 18.7% of cases. 39.3% of patients had Simpson I resection, 34.3% had Simpson II, 5.4% had Simpson III, 20.6% had Simpson IV, and 0.5% had Simpson V. The risk factors for incomplete resection were: symptomatic presentation (OR 0.56 [0.43–0.72]), skull-base location (OR 0.79 [0.70–0.88]), and bone invasion (OR 0.85 [0.73–0.99]). Using a recursive partitioning analysis, we propose a classification-tree for the prediction of GTR rate based on preoperatively determinable patient- and tumor characteristics. The identification of preoperative predictors of poor GTR rate may aid clinicians managing meningioma patients. In selected cases were the predicted GTR rate is low, staged treatment with surgical debulking followed by adjuvant therapy may be favored in order to minimize postoperative morbidity and mortality.

## Introduction

Meningiomas are generally considered histologically benign tumors that typically present as an intracranial extracerebral dural lesion with homogenous contrast enhancement on magnetic resonance imaging (MRI). Owned to an aging population and the increasing availability of imaging diagnostics, more incidental meningioma are detected, thus leading to a higher overall incidence^1,2^.

Besides surveillance, the therapeutic management of meningiomas is primarily surgical and aims at maximal tumor removal as the complete removal of tumor and its dural tail, which is important for later disease control, and to obtain a pathological diagnosis^3–5^. Depending on the size, location, and anatomical relationship of the tumor with the surrounding structures, achieving a complete resection can be challenging. The extent of resection (EOR) is quantified using the Simpson scale^6^. Several studies assessed EOR as a prognostic factor of overall and progression-free survival (OS and PFS)^7–11^, but none addressed specifically preoperative factors determining EOR. However, identification of predictive factors of surgical resection might be helpful for “personalized surgery”, i.e. in tailoring surgical resection on a case-by-case basis.

The aim of this study is to identify predictive factors of EOR and to propose an estimation of the predicted EOR based on patient’s and tumor’s characteristics.

## Results

### Patients characteristics

1469 patients (1033 females & 436 males) surgically treated for a meningioma were identified. The mean age at surgery was 64 ± 20.1 years. The female-to-male ratio was 2.4:1. The mean Karnofsky performance status (KPS) was 81.2 ± 12.1. Neurologic deficit was the most common presenting symptom (60.2%), followed by signs of intracranial hypertension (ICH) (31.7%) and seizures (29.6%). 5.4% of patients were asymptomatic. The mean follow-up was 7.8 ± 5.5 years (Table 1). One patient moved abroad and was lost to follow-up.

**Table 1:** Characteristics of a surgical population of patients with meningiomas (n = 1469).

	n	%
Age	64 ± 20.1	—
Sex	1033 F/436 M	—
Preoperative KPS	81.18 ± 12.1	—
Presenting symptoms		
Asymptomatic	79	5.4%
Seizures	435	29.6%
ICH	466	31.7%
Neurological deficit	855	60.2%
Skull base meningioma	690	47%
WHO grade		
I	1352	92.3%
II	77	5.2%
III	32	2.2%
Bone invasion	274	18.7%
Simpson grade		
I	575	39.2%
II	503	34.2%
III	79	5.4%
IV	302	20.6%
V	8	0.6%
GTR	1159	78.9%
Follow-up (years)	7.8 ± 5.5	—

GTR: Gross total resection; ICH: intracranial hypertension; KPS: Karnofsky performance score; WHO: World Health Organization.

### Tumors characteristics

47% of the meningiomas were skull base meningiomas. World Health Organization (WHO) grades were I for n = 1352 (92.3%), II in n = 77 (5.2%), and III for n = 32 patients (2.2%). Bone infiltration was described in n = 274 (18.7%) of the cases (Table 1).

### Extent of resection

Regarding EOR, n = 575 patients (39.3%) had Simpson I resection, n = 503 (34.3%) Simpson II, n = 79 (5.4%) Simpson III, n = 302 (20.6%) Simpson IV, and n = 8 (0.5%) Simpson V. GTR defined as a Simpson grade I, II or III resection^12^, was achieved in n = 1075 (79.3%) of surgeries (Table 1).

Skull base meningiomas were associated with higher Simpson grades than non skull-base meningiomas. This held true especially for anatomically difficult locations such as the orbit, the petroclival region or the cavernous sinus. As expected, GTR was more often achieved in convexity (96.7%) and lateral sphenoid wing (87.2%) meningiomas.

### Predictive factors of EOR

**Figure 1:**
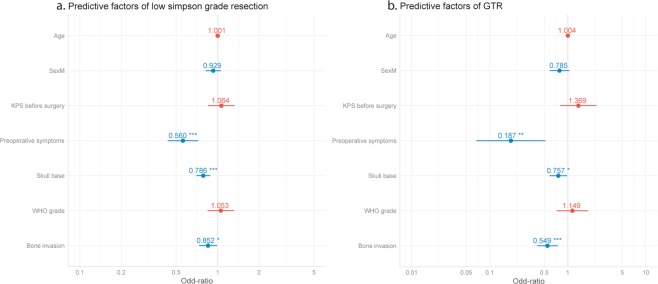
Forrest plots of predictive factors for meningioma surgical extent of resection. (**a**) Predictive factors of a good resection based on the Simpson grade. (**b**) Predictive factors of gross total resection (GTR).

Three independent risk factors for incomplete resection were identified: symptoms at presentation (seizure, intracranial hypertension and/or a neurological deficit) (OR 0.56 [0.43–0.72]), a skull base meningioma location (OR 0.79 [0.70–0.88]), and associated bone invasion (OR 0.85 [0.73–0.99]) (Table 2, Fig. 1). When considering the different modes of clinical presentation, GTR was 25% less likely in patients who had a preoperative neurological deficit and 33% more likely in patients presenting with preoperative seizures (Table 2). Age, sex, preoperative Karnofsky, WHO grade and preoperative intracranial hypertension did not have a significant impact on the EOR.

**Table 2:** Predictive factors of meningioma surgical extent of resection.

	Low Simpson grade		Gross Total Resection	
	OR	p-value	OR	p-value
Age	1.00 [0.99;1.01]	0.67	1.00 [0.99;1.01]	0.38
Sex (Male)	0.93 [0.82;1.06]	0.26	0.79 [0.58;1.05]	0.10
Preoperative KPS ≥ 70	1.06 [0.85;1.33]	0.58	1.37 [0.82;2.40]	0.25
Preoperative symptoms	0.56 [0.43;0.72]	**<0**.**001**	0.19 [0.06;0.46]	**<0**.**001**
Skull base meningioma	0.79 [0.70;0.88]	**<0**.**001**	0.76 [0.58;0.98]	**0**.**03**
WHO tumor grade	1.05 [0.84;1.31]	0.64	1.15 [0.84;1.31]	0.55
Bone invasion	0.85 [0.73;0.99]	**0**.**03**	0.55 [0.73;0.99]	**<0**.**001**

KPS = Karnofsky Performance Score. OR = Odds ratio. WHO = World Health Organization.

Odd-ratios (OR) represent the factor association with gross total resection (GTR: defined as Simpson 1, 2 or 3).

### Classification tree of EOR’s probability

**Figure 2:**
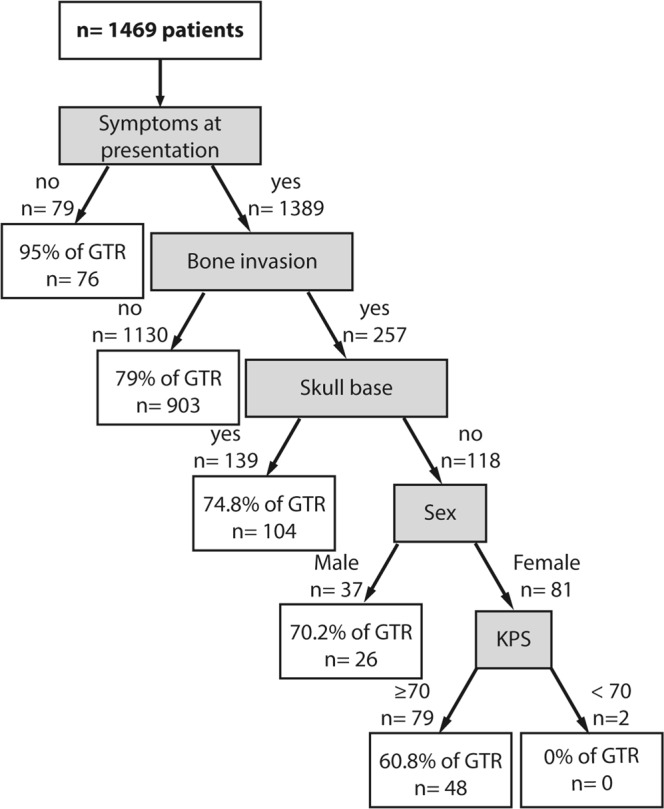
Classification tree of the preoperative predictors of meningioma’s extent of resection (EOR).

The identified preoperative predictors of meningioma EOR were put in hierarchical order using the recursive partitioning analysis with: the existence of symptoms at presentation, a tumor bone invasion, a skull-base located tumor, the patient’s sex and the preoperative KPS (Fig. 2). The GTR varies greatly between the groups from 95% for patients without a preoperative deficit to 60.8% for female patients with a preoperative deficit, a KPS < 70 and a skull base tumor with a bony infiltration, for example (Fig. 2).

## Discussion

The presence of symptoms at presentation, a skull-base location and/or a bone invasion were all negative predictive factors for GTR. To the best of our knowledge, this is the largest study analyzing predictive factors of the surgical EOR in meningiomas^9–11,13^. The preoperative KPS was not a significant predictive factor of EOR despite being predictive for postoperative neurological outcome, OS and PFS^14,15^. Age, preoperative KPS, and female-to-male ratio in our cohort were comparable to meningioma patients characteristics in the literature^5,11,13,16–18^. Similar to other series^11,19^, a Simpson grade I resection was achieved in 39.4% and a GTR in 78.9% of the cases.

Besides the presence of symptoms at presentation, the patients’ baseline characteristics were not predictive for EOR. The clinical presentation (seizure, ICH and/or a neurological deficit) was the sole patient-dependent factor identified as a significant factor of low EOR. This never previously described association may be explained by the surgeon’s apprehensiveness to aim for GTR in an already neurologically impaired patient. Increased GTR rates in patients with preoperative seizures may be linked to the meningioma’s location. Convexity and anterior skull base meningiomas are notoriously more epileptogenic, while also being more surgically accessible^20,21^.

Patient’s age was not a significant prognostic factor of EOR. Simpson I resection was generally attempted even in older patients, which is in line with previous studies^22^. Patients’ gender was not a significant independent prognostic factor in multivariate analysis, despite the increased incidence skull base meningiomas in women (female-to-male ratio 3.13:1, p < 0.001)^11,16^. In our study, the preoperative KPS was not a significant predictive factor of surgical resection’s quality despite being an important predictor of postoperative neurological outcome in several publications^14,15^.

Meningiomas with skull-base location and bone invasion were less often completely resected. This is unsurprising as these locations can be technically more challenging due to their restricted surgical access and vicinity to vascular and/or neurological structures^11,18,23,24^. This difference was particularly stark between convexity and cavernous or petroclival meningiomas, where a Simpson grade I or even grade II resection was rarely possible.

Bone invasion was another significant independent factor of poor resection quality. This infiltration requires additional drilling of the bone close to the dural insertion, often on the skull flap. In certain cases, bone invasion management represents a major part of the surgical procedure, for instance in spheno-orbital meningiomas where the extent of bone invasion and cavernous sinus involvement may not allow complete removal^25^.

This study is the first to propose a classification-tree of the predictors of EOR (Fig. 2). As can be seen from the first level of the tree, the EOR is very high for patients without a symptoms at presentation. Possibly, meningioma surgery for asymptomatic patients is usually more carefully planned and more likely proposed almost only if a complete resection can be performed. Going further down the tree, the presence of a bone invasion represents the second main predictor of low EOR, followed by the tumor location. This may be explained by the fact that these tumors are harder to remove completely while removing the bony infiltration. Note that the preoperative factors play only a minor role in predicting the EOR. However, this classification tree did not consider the meningioma’s radiological features, that are important for the preoperative planning. For example, the size of the lesion and the mass effect on the surrounding brain structures has an impact on the planned and observed EOR, even in asymptomatic patients.

Considering these factors may be helpful in the decision-making process and the planning process of the surgical resection. The proposed classification tree that allows for a rapid estimation of the estimated EOR in consideration of the patient and tumor preoperative characteristics. Although complete surgical resection while preserving the patient’s neurological status is the goal, a Simpson I resection is neither always attainable nor does it guarantee increased OS or PFS^9,11^. This holds especially true in view of other available adjuvant therapeutic options such as radiotherapy^26–28^, second surgery^29–31^, or a watchful *wait-and-scan* follow-up.

Recently, there has been a trend towards more conservative treatment for meningiomas, mainly because it has been shown that the Simpson grade is not universally applicable to all meningiomas^9,26,32^. Hence, a staged treatment with debulking followed by adjuvant treatment might be preferable in order to minimize postoperative morbidity and mortality, especially in skull-base meningiomas. The predictive factors of low EOR identified in this study may help neurosurgeons to identify those patients who may fare better with staged treatment^9,33^.

The main strengths of this clinical study are the clinical setting, the number of patients as well as the length of follow-up up to 21 years. Loss of follow-up was minimal since all patients with a postoperative complication or recurrence were systematically re-referred to our tertiary center. Only one patient was lost to follow-up, moving abroad. The data stem from one neurosurgical center with mostly homogenous surgical practices. This can make the generalization of our finding to all patients difficult. All patients with a histologically proven meningioma were included, which reduces selection bias. The retrospective data collection before 2003 is a limitation. Also, due to the long period of data collecting starting in 1990, radiological data, especially MRI, tumor size and molecular biomarkers such as Mib-1 or Ki67 were not available for all patients and were included in the statistical analysis.

The choice to regroup all meningiomas together for the statistical analysis and the data interpretation may also be subject to question as meningioma is not a homogenous pathology, with different pattern of evolution and therapeutic management for specific subgroups, especially depending of the location and WHO grade. The global results may not be representative of these specific categories of meningioma.

## Conclusions

Clinical symptoms at presentation, skull-base location and bone invasion were significant predictors of a poor EOR for meningiomas. The identification of these factors may aid clinicians managing patients with meningiomas.

## Methods

### Patient cohort

Data were acquired from a retrospectively (1990 to 2002) and prospectively managed (2003 to 2010) databank of Oslo University Hospital (OUH). OUH is the main Norwegian tertiary referral center and has two neurosurgical units (Rikshospitalet and Ullevaal), which covers an area of approximately three million inhabitants, i.e. 56% of the Norwegian population.

All patient operated for a meningioma during the study period at OUH were included. Preoperative imaging studies were reviewed to confirm tumor location and size, contrast enhancement, and presence of calcification. The WHO grading system was used to classify the histology of meningiomas. The WHO criteria changed during the study period. From 1990 to 2001, the tumors were classified as benign, atypical or anaplastic. The present WHO-grading system for meningioma was implemented in 2001, which divides the tumors into grade I, II and III. For this study, we reclassified the tumors operated before 2001 to the present WHO classification; benign = WHO grade I, atypical = WHO grade II and anaplastic = WHO grade III. The definition of skull base meningioma was based on Al-Mefty *et al*.^34^. All patients were operated on by the neurosurgical teams of OUH. The EOR was assessed using the Simpson grade scale, based on the surgical report in conjunction with post-operative imaging. Gross total resection (GTR) was defined as a Simpson grade I, II or III resection^12^. The histopathological diagnosis of meningioma was confirmed by experienced neuropathologists.

### Ethics

The study was regulated by the Personal Data Act/Personal Health Data Filing System Act and approved by the Data Protection Official at OUH (2017/5204). Informed consent was not required according to the Personal Data Act/Personal Health Data Filing System Act.

### Statistical analysis

Statistical analysis was performed using R v3.5.1 (https://www.r-project.org). The significant p-value threshold was defined at 0.05. Multivariate analysis was performed using a linear generalized model approach. The variable considered for the multivariate analysis were patient’s age, sex, preoperative Karnofsky prognostic scale (KPS) and clinical status, as well as the tumor location, WHO histopathological grade and the presence of a bone invasion.

The above-mentioned factors were used to build a classification of predictors of EOR using the classification and regression tree (CART) recursive partitioning analysis. The variables considered were patient’s age, sex, preoperative KPS and preoperative status, as well as tumor location and bone invasion. The generated CART tree was pruned by adjusting the complexity variable to minimize the estimated error in order to avoid data overfitting.
